# Supplementary material for: Identification of diabetic retinopathy lesions in fundus images by integrating CNN and vision mamba models
Source: PLoS One. 2025 Jan 28;20(1):e0318264. doi: 10.1371/journal.pone.0318264 (PMC11774367; doi:10.1371/journal.pone.0318264)
Supplement: Attachment — Submitted filename: Response to Editor .docx [file pone.0318264.s001.docx]

Dear Editor

Thank you so much for your constructive comments and valuable suggestions, which are of great importance for improving the quality of our manuscript. Following the suggestions, we modified the manuscript carefully. In this letter, we list our detailed responses to all your questions and suggestions.

**------------------------- Response to comments -------------------------**
1. It appears that your ORCiD iD has not been validated in your Editorial Manager account and we are unable to proceed until that step is complete.

To validate your ORCiD iD in Editorial Manager, please follow the steps below:

a. In your Editorial Manager account, please go to ‘Update my Information’ (in the upper left-hand corner of the main menu), and click on the Fetch/Validate link next to the ORCiD field.

b. This link will take you to the ORCiD site and allow you to create a new iD or authenticate a pre-existing iD in Editorial Manager.

For additional instructions, please watch the following video for a step-by-step demonstration: <https://www.youtube.com/watch?v=_xcclfuvtxQ>

**Answer:** Thank you so much. I have updated my information with the ORCiD in my Editorial Manager account.


2. We note that your manuscript is not formatted using one of PLOS ONE’s accepted file types. Please reattach your manuscript as one of the following file types: .doc, .docx, .rtf, or .tex (accompanied by a .pdf).
If your submission was prepared in LaTex, please submit your manuscript file in PDF format and attach your .tex file as “other.”

**Answer:** Thank you so much. I have provided both the tex files and pdf files of the manuscript and the manuscript with tracked changes in the submission system.

3. We note that your author list was updated during the revision process. Please complete our Authorship Change Form attached. If you are adding or removing more than 2 authors, you can complete multiple forms and submit as many forms as needed to reflect the updates. Please return the form(s) as an attachment by emailing plosone@plos.org or by uploading it as a submission file labeled with the file type ‘Other’. Please note that if your manuscript is accepted, we will not be able to complete the publication process without the completed form.

**Answer:** Thank you so much. We have provided the author change form for Dr. Liu and Dr. Gao and submitted this form as ‘Other’ in the submission system.

4. We note that the links provided for the datasets used are not working. At this time, please provide working links or DOI to the dataset.

**Answer:** Thank you so much. We have double-checked the data availability statement in the revised manuscript as “The RFMiD 2.0 dataset, used in this study, is publicly available and can be accessed without any restrictions. The dataset is hosted on the Grand Challenges in Biomedical Imaging Platform, and interested researchers can download it from the following link: https://riadd.grand-challenge.org/Download/. Readers are advised to create an account on https://riadd.grand-challenge.org/Home/ then access the 'Download' tab https://riadd.grand-challenge.org/Download/, and the dataset can be downloaded by clicking on 'Training Set', 'Evaluation Set', and 'Test Set' links inside this webpage. And the DOI of this dataset is 10.3390/data6020014.

We have also used the public Kaggle dataset in this study. This dataset was considered in the Asia Pacific Tele-Ophthalmology Society (APTOS) 2019 blindness detection competition https://www.kaggle.com/datasets/mariaherrerot/aptos2019. Readers are advised to create an account on Kaggle. And the dataset folder can be downloaded by clicking on 'Download' button inside this webpage.

The dataset Messidor for this study can be found in the website, https://www.adcis.net/en/third-party/messidor, and its DOI is 10.5566/ias.1155. A form with personal information needs to be completed to download this dataset. Readers are advised to download this form by clicking on 'Download form' button inside this webpage.”.

To note that the readers might need to create accounts or fill up a form before they can download these three datasets. We have provided the addresses for them to create user account or download the form.

-------------- Reviewer #1 Comments ------------------------

Reviewer #1:This article introduces a novel deep learning model that integrates convolutional neural networks (CNNs) with Vision Mamba models to accurately detect and classify diabetic retinopathy lesions in fundus images. The proposed methodology demonstrates superior performance compared to advanced algorithms on publicly available datasets, making it a valuable tool in therapeutic applications. The manuscript is engaging, well-structured, and well-written. To further enhance its quality and facilitate processing, the following suggestions are provided:

1.Elaborate on the specific limitations of previous studies that the proposed model addresses. This will clearly position the current work as a significant contribution to the field.

**Answer:** Thank you so much for pointing this out. Following your suggestion, we have modified the introduction section in the revised manuscript by adding the following description “CNN-based models can offer robust feature extraction capabilities, operate efficiently due to parameter sharing and sparse connectivity, and exhibit translational invariance, making them highly effective for image processing tasks. However, they have limitations in capturing global image dependencies, may struggle with transformations such as rotation and scaling, require substantial computational resources, and depend heavily on large annotated datasets for training, which can be a challenge in scenarios with limited data availability.” and “ViT models have emerged as a powerful alternative to CNNs in the field of computer vision, offering several advantages such as the ability to capture long-range dependencies and process inputs of varying sizes, which enhances their flexibility and potential for greater generalization. They have demonstrated superior performance on standard datasets, showcasing their effectiveness in image classification and other vision tasks. However, ViTs also come with significant challenges, primarily due to their high computational and memory costs associated with the self-attention mechanism, especially when dealing with high-resolution images. This quadratic computing cost can be prohibitive for real-time applications and large-scale deployments. Additionally, ViTs often require substantial labeled data to achieve optimal performance, which can be a limiting factor in certain scenarios.”.

2.To provide a more comprehensive overview of the research landscape, incorporate recent related works (2021–2024) into the literature review. Including the following works would be beneficial:

https://www.sciencedirect.com/science/article/abs/pii/S1746809424008358, https://ieeexplore.ieee.org/abstract/document/10559800

**Answer:** Thank you so much for pointing this out. Following your suggestion, we have added more works and the above-mentioned works in the literature review of the revised manuscript as Pandey and Kumar \cite{Pandey2024} proposed a cascade network using lightweight CNN and CNN Xception network to perform binary classification and multi-grading of DR and diabetic macular edema (DME). Recently, Abushawish \etal \cite{Abushawish2024} presented a survey of the evolution in deep learning models for DR detection, focusing on the transition from machine learning to deep learning algorithms such as CNNs.”.

3.The statement, "The suggested Vision Mamba component has equivalent power to ViT while requiring substantially less computational complexity," requires further elaboration. Include a detailed explanation and supporting information to substantiate this claim.

**Answer:** Thank you so much for pointing this out. A significant feature of Vision Mamba is that it does not rely on traditional attention mechanisms while maintaining comparable modeling capabilities to ViT. Specifically, the computational complexity of ViT's self-attention mechanism is a quadratic function of the sequence length M, i.e., $4MD^{2}+2M^{2}D$.In contrast, the computational complexity of the State Space Model (SSM) in VMamba is a linear function of the sequence length M, i.e., $3M(2D)N+M(2D)N$,where N is a fixed parameter, defaulting to 16.This indicates that for long sequences, VMamba's computational complexity is significantly lower than that of ViT.

Following your suggestion, we modified the contributions of this work into “The vision mamba component, which is central to the proposed pipeline, demonstrates a performance parity with ViT in terms of accuracy but with a significant reduction in computational complexity. This advancement is crucial for applications where resource constraints are prevalent, such as in mobile health diagnostics.” in the revised manuscript. In addition, we added more description about the complexity comparison as “To note that the computational complexity of ViT's self-attention mechanism is a quadratic function of the sequence length M, i.e., $4MD^2+2M^2D$. In contrast, the computational complexity of the SSM in vision mamba is a linear function of the sequence length M, i.e., $3M(2D)N+M(2D)N$, where N is a fixed parameter, defaulting to 16. This indicates that for long sequences, vision mamba's computational complexity is significantly lower than that of ViT. ” in the revised manuscript.

1. Enhance the clarity and impact of findings by including graphical representations such as accuracy and loss curves for both training and testing phases. Additionally, provide fundus image with classifications results based on retinopathy grades.

**Answer:** Thank you so much for pointing this out. Following your suggestion, we added the accuracy and loss curves for both training and testing phases (as shown in Figure 4 and Figure 6). In addition, we also provide the fundus images with classification results in the revised manuscript (as shown in Figure 8).

5. Write the literature survey in past tense to maintain a consistent and professional tone.

**Answer:** Thank you so much for pointing this out. Following your suggestion, we modified the literature survey in past tense in the revised manuscript.

6. The statement, “An end-to-end training approach was used to train the proposed model by partitioning the complete OATH dataset into a training set (80%) and a testing set (20%),” lacks clarity regarding the OATH dataset. The authors should describe this dataset in detail, as only RFMiD 2.0, APTOS2019, and Messidor datasets are mentioned elsewhere in the manuscript.

**Answer:** Thank you so much for pointing this out and we are sorry for the confusion. This dataset was used in our other study and not used in this work. Following your suggestion, we have removed this description in the revised manuscript.

7.Include a comparison of the proposed method with recent works (2021–2024) to better contextualize its contributions.

**Answer:** Thank you so much for pointing this out. Following your suggestion, we have added the following comparison in the revised manuscript as “Compared to our method, their work focused on the integration of optimization algorithms, while our approach emphasizes structural innovation of the model and depth of feature extraction.”, “Our model can surpass their cascade network in terms of feature extraction and classification accuracy.”, “Our work complements their research by further enhancing CNNs by integrating vision mamba model to improve the accuracy of DR detection.”, “Compared to Halder's work, our study not only focuses on model performance but also on the interpretability and practical clinical application of the model.”, “Our model, while integrating these technologies, also introduces additional innovations such as adaptive feature fusion and multi-scale analysis to further improve classification accuracy.”, and “Our model extends beyond ViT by introducing novel attention mechanisms to improve the recognition of subtle pathological features.”.

8. The discussion of results should be more comprehensive. Include deeper insights, interpretations, and implications to strengthen the manuscript's impact.

**Answer:** Thank you so much for pointing this out. Following your suggestion, we have modified the whole discussion section in the revised manuscript.

9. Revise and enhance the quality of the figures.

**Answer:** Thank you so much for pointing this out. Following your suggestion, we have modified the figures in the revised manuscript.

-------------- Reviewer #2 Comments ------------------------

Reviewer #2: Major Concerns:

1. The manuscript claims to present a novel framework combining CNN and Vision Mamba models, but the explanation of the methodology is vague and lacks sufficient detail. The Vision Mamba model's specifics and how it integrates with CNNs are not sufficiently described or justified.

**Answer:** Thank you so much for pointing this out. Following your suggestion, we have modified the methodology section and provided three separate subsections to describe the CNN, Vision Mamba, and the integrated model in the revised manuscript.

2. The authors fail to provide clear novelty over existing state-of-the-art models. Many components, such as the use of Inception-ResNet and bidirectional state-space mechanisms, appear to be incremental rather than novel advancements.

**Answer:** Thank you so much for pointing this out. Following your suggestion, we have added the novelty comparison over the state-of-the-art methods in the introduction section of the revised manuscript as “Compared to our method, their work focused on the integration of optimization algorithms, while our approach emphasizes structural innovation of the model and depth of feature extraction.”, “Our model can surpass their cascade network in terms of feature extraction and classification accuracy.”, “Our work complements their research by further enhancing CNNs by integrating vision mamba model to improve the accuracy of DR detection.”, “Compared to Halder's work, our study not only focuses on model performance but also on the interpretability and practical clinical application of the model.”, “Our model, while integrating these technologies, also introduces additional innovations such as adaptive feature fusion and multi-scale analysis to further improve classification accuracy.”, and “Our model extends beyond ViT by introducing novel attention mechanisms to improve the recognition of subtle pathological features.”.

In addition, we have also modified the contributions of this study in the introduction section of the revised manuscript as “

We introduce a pioneering pipeline that synergizes CNN with a vision mamba for the precise identification of DR lesions. This integration is innovative as it leverages the strengths of CNNs in feature extraction and the efficiency of vision mamba in processing visual data.

The vision mamba component, which is central to the proposed pipeline, demonstrates a performance parity with ViT in terms of accuracy but with a significant reduction in computational complexity. This advancement is crucial for applications where resource constraints are prevalent, such as in mobile health diagnostics.

The experimental findings indicate that the proposed model surpasses existing state-of-the-art algorithms in both the detection and grading of DR. This achievement is pivotal as early and accurate diagnosis is essential for timely intervention and can potentially prevent vision loss among diabetic patients.”.

3. The dataset usage and preprocessing steps are inadequately described. For example, the partitioning of datasets for training and testing lacks clarity on whether overlapping patients were excluded to prevent data leakage.

**Answer:** Thank you so much for pointing this out. Following your suggestion, we have revised our methodology to explicitly state that we have taken measures to exclude overlapping patients between training and testing datasets to prevent data leakage. In addition, we have provided the description of “In this study, we have meticulously partitioned the entire dataset to ensure that the training (80\%) and testing set (70\%) are devoid of any overlapping patient data, which is a critical step in preventing data leakage. We acknowledge the importance of maintaining the sanctity of our model's evaluation process and have thus implemented stringent measures to preclude the inclusion of the same patient data in both sets. This meticulous exclusion is pivotal in upholding the impartiality of our model's performance assessment and ensures that the generalization capabilities of our model are accurately represented.” in the revised manuscript.

4. Hyperparameter tuning details and experimental controls to ensure reproducibility are insufficiently detailed.

**Answer:** Thank you so much for pointing this out. Following your suggestion, we have modified the hyperparameter tuning details and experimental controls in the revised manuscript as “Additionally, we have implemented a training strategy that includes a careful selection of hyperparameters, such as learning rate, batch size, and the number of epochs. The learning rate determines the step size at each iteration while moving toward a minimum of a loss function, and we have chosen an optimal value that allows for effective convergence without causing divergence. The batch size and the number of epochs were selected based on early stopping to prevent overfitting while ensuring the model trains until convergence. ”.

5. The manuscript does not adequately address the potential for bias introduced by the imbalance in diabetic retinopathy grades across datasets.

**Answer:** Thank you so much for pointing this out. We have adjusted the class weights during model training to give more importance to the minority classes. This approach helps in reducing the bias towards the majority classes and improves the model's ability to classify under-represented classes accurately. We have also chosen evaluation metrics that are sensitive to class imbalance, such as precision, recall, F1-score, and area under the ROC curve (AUC-ROC). These metrics provide a more comprehensive assessment of the model's performance across all classes, not just the majority class. Following your suggestion, we added the description of “To note that there is potential for bias introduced by the imbalance in diabetic retinopathy grades within the used datasets. To mitigate this, we have implemented several strategies to ensure the robustness and fairness of our model's performance, including:

Class Weight Adjustment: We have incorporated class weight adjustments during model training to counteract the disproportionate representation of classes, thereby enhancing the model's sensitivity to minority classes.

Comprehensive Evaluation Metrics: We have selected a suite of evaluation metrics that are less sensitive to class imbalance, including precision, recall, F1-score, and AUC-ROC, to provide a balanced assessment of our model's performance across all classes.

Data Augmentation: To enrich the dataset and reduce the impact of class imbalance, we have applied data augmentation techniques to the minority classes, creating a more diverse and representative training set. ” in the revised manuscript.

6. The results are compared with an extensive list of methods, but the comparisons lack statistical rigor. There is no mention of significance testing or confidence intervals to substantiate claims of superior performance.

**Answer:** Thank you so much for pointing this out. Following your suggestion, we have performed the ANOVA test and the statistical analysis has revealed that the differences of performance metrics between the proposed approach and the state-of-the-art methods are statistically significant (p < 0.05). In the revised manuscript, we have added the description of “The statistical analysis has revealed that the differences in performance metrics between the proposed approach and the state-of-the-art methods are statistically significant (p < 0.05). ”.

7. The choice of metrics, while standard, does not include sufficient consideration for imbalanced datasets beyond weighted metrics.

**Answer:** Thank you so much for pointing this out. We appreciate your feedback on the need for additional metrics that consider the imbalance in our datasets. Based on the current standards and best practices for evaluating models on imbalanced datasets, we have selected a set of metrics that provide a comprehensive evaluation, including weighted F1 score (wF1), weighted Kappa (wKappa), F1 score, sensitivity (recall), precision, and AUC-ROC. Here's why these metrics are suitable for imbalanced datasets:

Weighted F1 Score (wF1): The F1 score is the harmonic mean of precision and recall, providing a balance between these two metrics. By applying weights, wF1 accounts for the class imbalance, giving more importance to the minority class.

Weighted Kappa (wKappa): Kappa statistic is a measure of inter-rater agreement that accounts for the agreement occurring by chance. Weighted Kappa adjusts this measure to account for the imbalance in class distribution, making it a more accurate reflection of model performance on imbalanced datasets.

Sensitivity (Recall): This metric focuses on the proportion of actual positive cases that are correctly identified. It is particularly important in imbalanced datasets where the minority class is often the class of interest.

Precision: Precision measures the proportion of positive identifications that were actually correct. It is crucial in cases where false positives can be as costly as false negatives, which is often the case in imbalanced datasets.

AUC-ROC: The Area Under the Receiver Operating Characteristic Curve provides a measure of the model's ability to distinguish between classes. It is less sensitive to class imbalance than accuracy and can provide a more comprehensive view of model performance across all thresholds.

These metrics, when used together, provide a well-rounded assessment of model performance on imbalanced datasets. They allow us to evaluate not only the overall performance but also the model's ability to correctly identify the minority class, which is often the primary concern in such scenarios.

8. Figures such as the framework diagram (Fig. 1) are not sufficiently annotated, making it difficult to understand the pipeline without referring extensively to the text.

**Answer:** Thank you so much for pointing this out. Following your suggestion, we have modified the figures in the revised manuscript.

9. Confusion matrices presented are inadequately analyzed, with no discussion of patterns in misclassification or how these errors relate to clinical significance.

**Answer:** Thank you so much for pointing this out. Following your suggestion, we have added the description about the outcomes of the confusion matrices in the result section of the revised manuscript as “These results in \myfigure{fig:confusion1} indicate that the model performs well in identifying both healthy and DR cases, with high accuracy, precision, recall, and F1 scores. However, it is also important to note that despite the model's overall promising performance, a small number of healthy individuals were incorrectly predicted as DR (53 false positives) and a small number of DR individuals were incorrectly predicted as healthy (50 false negatives).” and “The confusion matrix as shown in\myfigure{fig:confusion2} shows the number of instances for each actual DR grade (Ground Truth) that were predicted to be each of the possible grades (Predicted label):

DR0: The model correctly predicted 1717 instances as DR0 (True Negatives for DR0). However, it misclassified 30 instances of DR1, 27 of DR2, 18 of DR3, and 13 of DR4 as DR0 (False Negatives for those grades, respectively).

DR1: For DR1, the model correctly predicted 311 instances (True Positives for DR1). It had 31 misclassifications from DR0, 12 from DR2, 8 from DR3, and 8 from DR4.

DR2: The model accurately predicted 915 instances as DR2 (True Positives for DR2), which is a strong performance for this grade. It had 25 misclassifications from DR0, 14 from DR1, 9 from DR3, and 36 from DR4.

DR3: For DR3, the model correctly predicted 150 instances (True Positives for DR3). It had 22 misclassifications from DR0, 8 from DR1, 13 from DR2, and none from DR4.

DR4: The model correctly predicted 228 instances as DR4 (True Positives for DR4). It had 10 misclassifications from DR0, 7 from DR1, 32 from DR2, and 18 from DR3.”.

10. The discussion section lacks depth in interpreting the findings. For example, the authors do not explore the implications of their model's limitations in real-world settings or address the constraints of high computational costs for deploying Vision Mamba in clinical environments.

**Answer:** Thank you so much for pointing this out. Following your suggestion, we have modified the discussion section as “Finally, we acknowledge that while the proposed CNN-Vision Mamba model demonstrates excellence in capturing feature embeddings and detecting global interconnections within retinal images, there are limitations that must be considered for real-world deployment. For instance, the model's reliance on high-quality, positional information may be challenged by variations in image acquisition across different clinical settings. Additionally, while we have optimized for computational efficiency, the practical constraints of clinical environments, such as the availability of processing power and memory, may impact the model's scalability and speed of execution.” in the discussion section of the revised manuscript.

11. Several claims, such as the "equivalence of Vision Mamba to ViT with lower computational cost," are made without empirical evidence or references.

**Answer:** Thank you so much for pointing this out. Following your suggestion, we have added the description about the comparison between Vision Mamba and ViT as “To note that the computational complexity of ViT's self-attention mechanism is a quadratic function of the sequence length M, i.e., $4MD^2+2M^2D$. In contrast, the computational complexity of the SSM in vision mamba is a linear function of the sequence length M, i.e., $3M(2D)N+M(2D)N$, where N is a fixed parameter, defaulting to 16. This indicates that for long sequences, vision mamba's computational complexity is significantly lower than that of ViT.” in the revised manuscript.

12. The ethics statement is marked as "N/A," which is inappropriate for a study involving human-related medical images. Ethical approval for dataset use should be explicitly stated.

**Answer:** First and foremost, we would like to express our gratitude for your valuable feedback. Your attention to the ethical considerations in our study involving human-related medical images is indeed crucial. In response to your concerns, we have made the following additions and corrections: (1) We confirm that the ophthalmic images used in our study are derived from public datasets that have undergone ethical review prior to their release. Each dataset employed in our research adheres to stringent ethical guidelines, including but not limited to obtaining informed consent, anonymization of data, and compliance with privacy protection regulations. (2) We ensure that all medical image data used in our study have been anonymized to protect patient privacy. This includes the removal of all identifiable information such as names, hospital names, etc., and all images have been processed to ensure that individuals cannot be recognized. (3) Informed Consent: Since we are using images from public datasets, these images were collected with the patients' informed consent, allowing their use in medical research.

13. The authors claim that datasets are publicly available but do not clarify their usage rights or ensure compliance with data-sharing standards.

**Answer:** Thank you for your insightful comments regarding the data usage rights and compliance with data-sharing standards in our study. We understand the importance of clarity on these matters, especially for a journal like PLOS ONE that has stringent policies on data availability and ethical research standards. (1) Data Availability Statement: We have revised our Data Availability Statement to explicitly mention the public repositories where the datasets are housed and the conditions under which they can be accessed. We have also included digital object identifiers (DOIs) for the datasets, as required by PLOS ONE. (2) Compliance with PLOS ONE Data Policy: We acknowledge PLOS ONE's requirement that all data necessary to replicate our study's findings must be publicly available without restriction at the time of publication. We have confirmed that our datasets meet this criterion and have provided the necessary information in our Data Availability Statement.

Minor Concerns:

The manuscript contains numerous grammatical errors and awkward phrasing, which detracts from readability.

• Original: "The proposed framework achieving state-of-the-art performance in diabetic retinopathy detection."

Issue: Missing verb ("is achieving" or "achieves") makes the sentence incomplete.

Revised: "The proposed framework achieves state-of-the-art performance in diabetic retinopathy detection."

• Original: "The dataset used for training were preprocessed to remove noise."

Issue: Subject-verb agreement error ("dataset" is singular but "were" is plural).

Revised: "The dataset used for training was preprocessed to remove noise."

• Original: "These results indicates the superiority of our model, as the robustness is validated."

Issue: Incorrect subject-verb agreement ("results" is plural but "indicates" is singular).

Revised: "These results indicate the superiority of our model, as its robustness is validated."

• Original: "In clinical setup, the proposed method can be easily implemented for its efficiency."

Issue: Awkward phrasing ("in clinical setup" should be "in a clinical setup").

Revised: "In a clinical setup, the proposed method can be easily implemented due to its efficiency."

And so on…

**Answer:** Thank you so much for pointing these out. Following your suggestion, we have double-checked the manuscript to eliminate the grammars and typos.
